# Supplementary material for: Reconstitution of the complete rupture in musculotendinous junction using skeletal muscle-derived multipotent stem cell sheet-pellets as a “bio-bond”
Source: PeerJ. 2016 Jul 19;4:e2231. doi: 10.7717/peerj.2231 (PMC4957990; doi:10.7717/peerj.2231)

| \| OP group \| OP \| OP \| Cont \| Cont \| OP \| Cont \| \| --- \| --- \| --- \| --- \| --- \| --- \| --- \| \| recovery term \| tension (g) \| N (1x10^2^) \| tension (g) \| N (1x10^2^) \| muscle mass (mg) \| muscle mass (mg) \| \| 17.5 \| 54.9 \| 53.802 \| 89.2 \| 87.416 \| 49.8 \| 51.2 \| \| 15 \| 34.9 \| 34.202 \| 89.4 \| 87.612 \| 36.2 \| 50.6 \| \| 16 \| 47.3 \| 46.354 \| 89.4 \| 87.612 \| 40.1 \| 50.6 \| \| 14 \| 15.8 \| 15.484 \| 41.2 \| 40.376 \| 29.6 \| 49.3 \| \| 5 \| 33 \| 32.34 \| 87.7 \| 85.946 \| 38.5 \| 54.9 \| \| 6.5 \| 32.1 \| 31.458 \| 52.3 \| 51.254 \| 28.4 \| 34.8 \| \| 8 \| 32.7 \| 32.046 \| 46.3 \| 45.374 \| 35 \| 46 \| \| 4 \| 6.3 \| 6.174 \| 42.99 \| 42.1302 \| 13.9 \| 52.3 \|  \| Control group \|  \|  \|  \| \| --- \| --- \| --- \| --- \| \| body mass \| muscle mass (g) \| tension (g) \| N (1x10^2^) \| \| 25 \| 50.8 \| 87.0 \| 85.26 \| \| 32 \| 51.7 \| 69.7 \| 68.35079 \| \| 33 \| 53.9 \| 76.8 \| 75.26037 \| \| 40 \| 50 \| 86.6 \| 84.8289 \| \| 41 \| 51.4 \| 86.7 \| 84.97208 \| |  |  |  |  |  |  |
| --- | --- | --- | --- | --- | --- | --- | --- | --- | --- | --- | --- | --- | --- | --- | --- | --- | --- | --- | --- | --- | --- | --- | --- | --- | --- | --- | --- | --- | --- | --- | --- | --- | --- | --- | --- | --- | --- | --- | --- | --- | --- | --- | --- | --- | --- | --- | --- | --- | --- | --- | --- | --- | --- | --- | --- | --- | --- | --- | --- | --- | --- | --- | --- | --- | --- | --- | --- | --- | --- | --- | --- | --- | --- | --- | --- | --- | --- | --- | --- | --- | --- | --- | --- | --- | --- | --- | --- | --- | --- | --- | --- | --- | --- | --- | --- | --- | --- | --- | --- | --- | --- | --- | --- | --- |

| RT-PCR 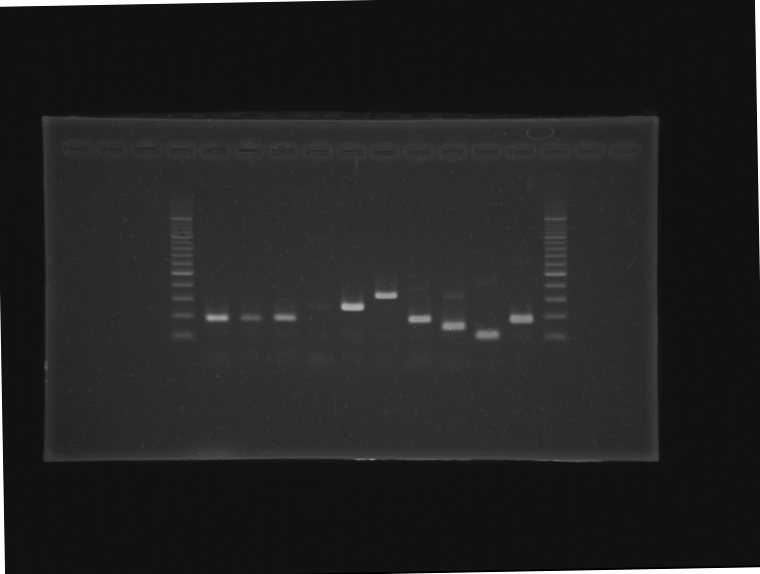 |  |  |  |  |  |  |
| --- | --- | --- | --- | --- | --- | --- |
| 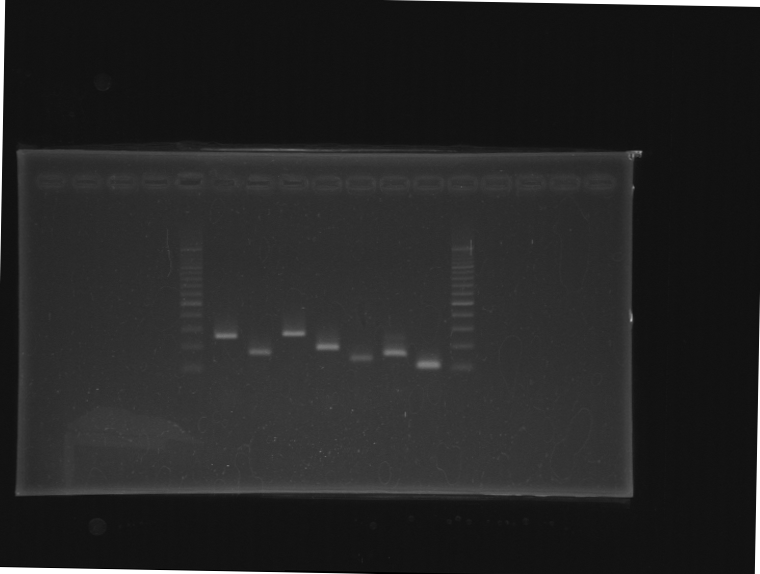 |  |  |  |  |  |  |
| 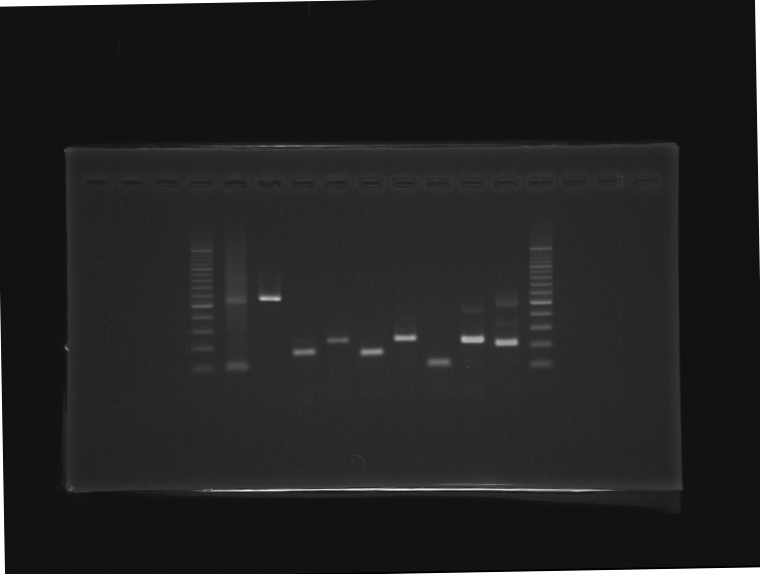 |  |  |  |  |  |  |
|  |  |  |  |  |  |  |
|  |  |  |  |  |  |  |
|  |  |  |  |  |  |  |
|  |  |  |  |  |  |  |
|  |  |  |  |  |  |  |
|  |  |  |  |  |  |  |
|  |  |  |  |  |  |  |
|  |  |  |  |  |  |  |
|  |  |  |  |  |  |  |


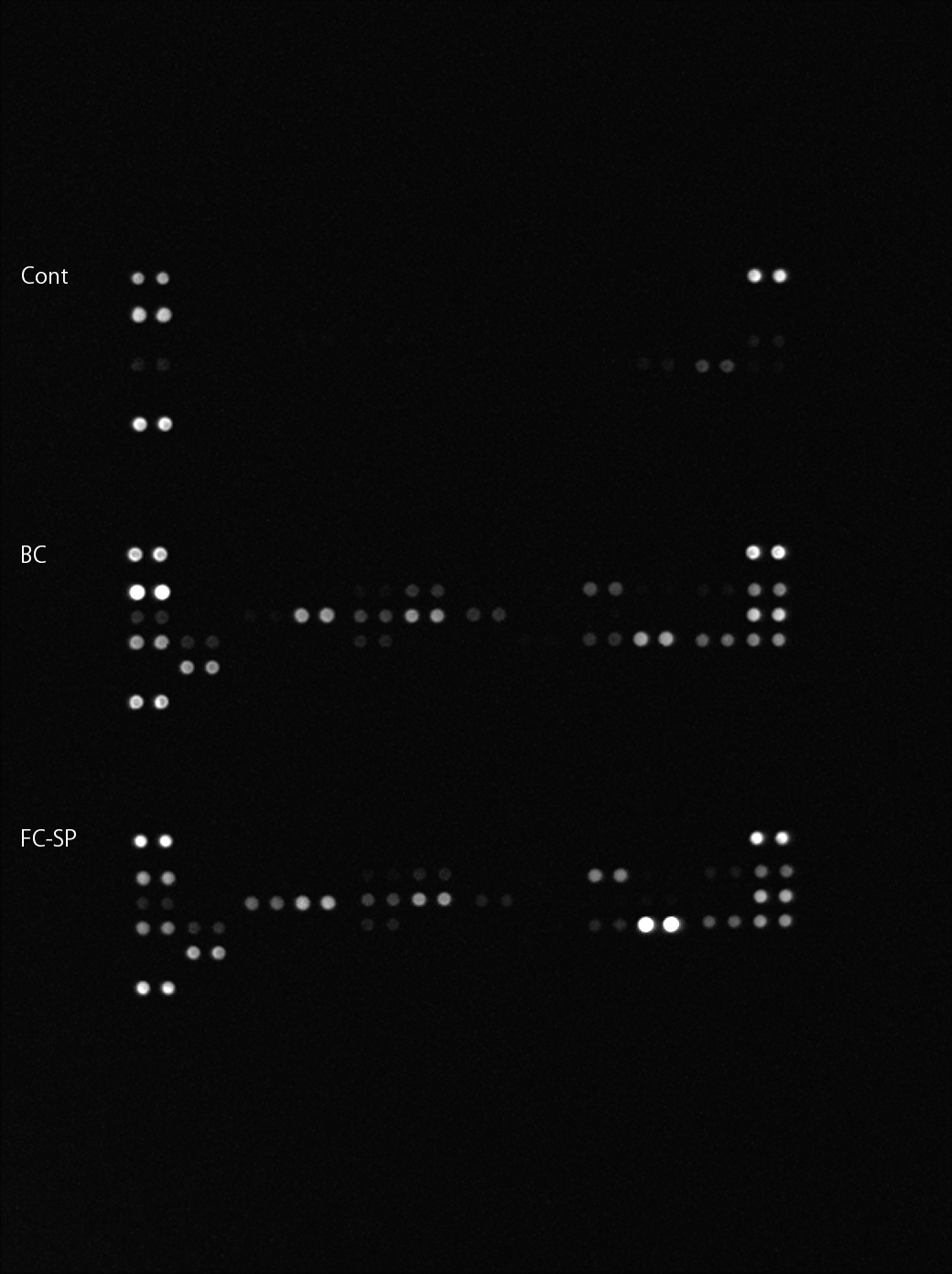

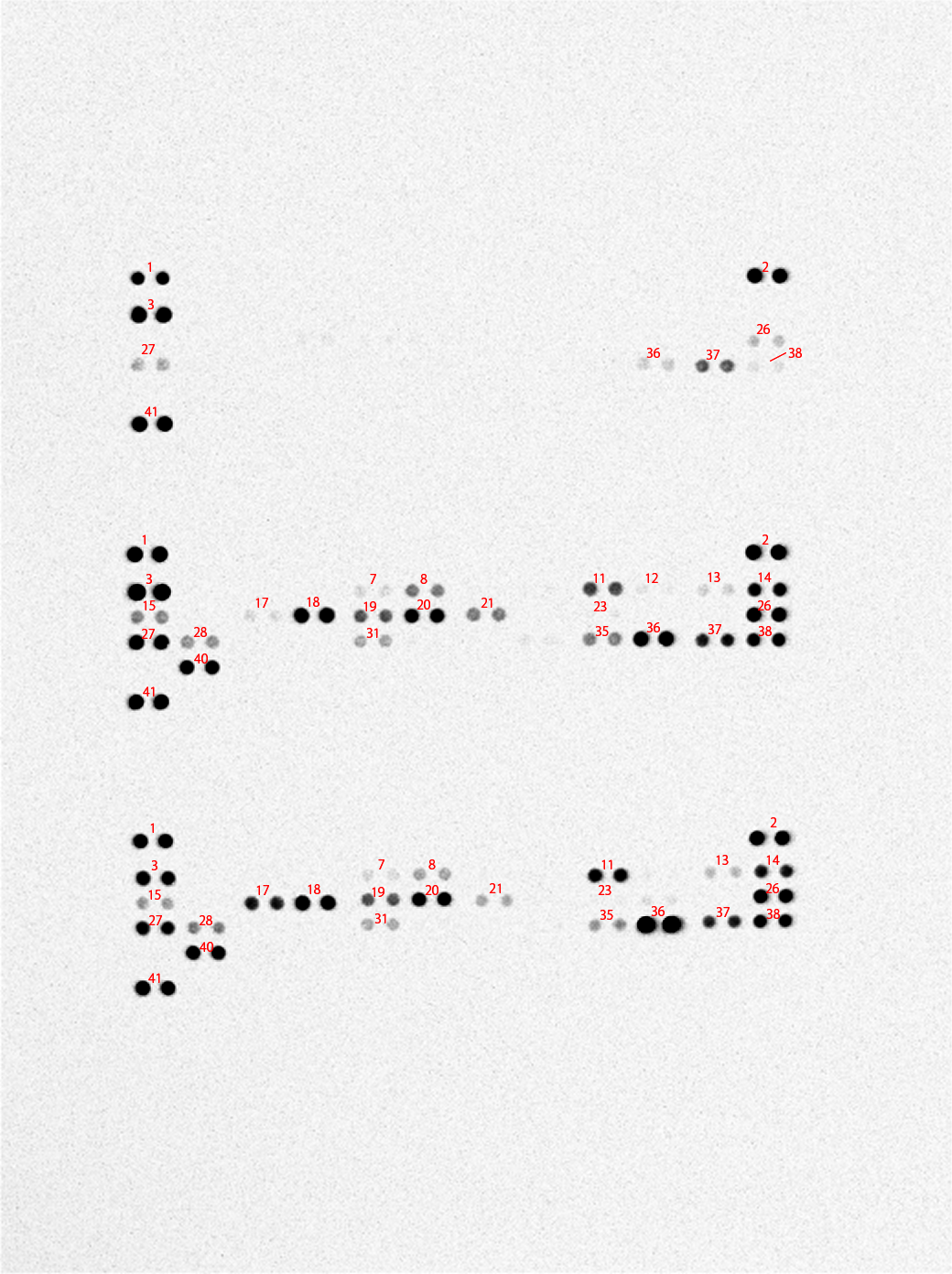

Supplement: Data S1 [file peerj-04-2231-s001.docx]
