# Supplementary material for: Reconstitution of the complete rupture in musculotendinous junction using skeletal muscle-derived multipotent stem cell sheet-pellets as a “bio-bond”
Source: PeerJ. 2016 Jul 19;4:e2231. doi: 10.7717/peerj.2231 (PMC4957990; doi:10.7717/peerj.2231)
Supplement: Table S1 — Specific primers and the analyzed materials are summarized. [file peerj-04-2231-s002.docx]

| Supplementary Table S1. Specific primers for mice. | | | | | |
| --- | --- | --- | --- | --- | --- |
| No | Gene name (full name and/or typical role) | product size (bp) | Forward primer | Reverse primer | Utilization purpose |
| 1 | **MyoD** (myogenic regulatory factors) | 184 | GGCCACTCAGGTCTCAGGTGT | TGTTGCACTACACAGCATGCCT | Myogenic determination and differentiation markers |
| 2 | **Myf5** (myogenic regulatory factors) | 179 | TTAGCAAACCATGAACACGAAACA | AAGGGGGCTTCATTTACCAGG |  |
| 3 | **Pax7** (paired box transcription factors, also known as satellite cell marker) | 192 | CCCAACAGGTTTTCCCAACTG | CGGCCTTCTTCTAGGTTCTGCT |  |
| 4 | **Pax3** (paired box transcription factors relate to embryonic muscle development) | 256 | TGGACAGTCTGCCCACATCTCAGC | GGGAGCCTGTGCTGTAGCAATCAG |  |
| 5 | **Myogenin** (myogenic regulatory factors) | 262 | TACGTCCATCGTGGACAGCAT | TCAGCTAAATTCCCTCGCTGG |  |
| 6 | **c-met** (hepatocyte growth factor receptor, which is present in quiescent satellite cell) | 337 | CCAAGCCGCGTATGTCAGTAAA | AATAAGTCGACGCGCTGCA |  |
| 7 | **M-cad** (M-cadherin, a Ca2+-dependent cell adhesion molecule which is present in quiescent satellite cell) | 193 | GGGCTCTCTCTTGGGATGTG | CTTCTGCACTCTGCCAGGAC |  |
| 8 | **MyH** (skeletal muscle myosin heavy chain, cell differentiation marker) | 152 | TCAGGAAAGCCCAGCATGAG | TGCACCAGGAGGTCTTGCTC |  |
| 9 | **Desmin** (Muscle relate intermediate filament protein, cell differentiation marker) | 106 | AGGGTCAGGACCGAGTTTGTG | GCCATGAGGGCAGTTTTCAG |  |
| 10 | **IGF1** (Insulin-like growth factor-1) | 185 | CTGCTTGCTCACCTTCACCAG | TCCGGAAGCAACACTCATCC |  |
| 11 | **NGF** (Nerve growth factor, peripheral nerve growth and trophic factor) | 106 | TGCACCACGACTCACACCTTC | TCCTGCTGAGCACACACACAC | Peripheral nerve growth and trophic factors |
| 12 | **BDNF** (Brain-derived neurotrophic factor) | 582 | GGGACTCTGGAGAGCGTGAAT | CCTTATGAATCGCCAGCCAAT |  |
| 13 | **GDNF** (Glial cell-derived neurotrophic factor, peripheral nerve growth and trophic factor) | 180 | GTGAATCGGCCGAGACAATG | CACACCGTTTAGCGGAATGC |  |
| 14 | **CNTF** (Ciliary neurotrophic factor, peripheral nerve growth and trophic factor) | 241 | TTTCTGCCTTCGCCTACCAG | TTGGCCCCATAATGGCTCTC |  |
| 15 | **LIF** (Leukemia inhibitory factor, peripheral nerve growth and trophic factor) | 169 | ATCGGATGGTCGCATACCTG | CCCACACGGTACTTGTTGCAC |  |
| 16 | **Ninjurin** (nerve injury-induced protein) | 243 | GGAGCAGGGCAATGATTTCG | GCCACGTCCATTACAGGCTTC |  |
| 17 | **Galectin-1** (Initial axonal growth regulator in peripheral nerves after axotomy) | 109 | TGTCTCAAAGTTCGGGGAGAGG | GGGCATTGAAGCGAGGATTG |  |
| 18 | **Nestin** (Nerve relate intermediate filament protein, cell differentiation marker) | 233 | GCTCTGGGCCAGCACTCTTAG | TGTAGACAGGCAGGGCTAGCA |  |
| 19 | **Sox10** (transcription factor relate to Schwann cell-development) | 213 | TCCCCATGTTCTTCCCATCC | CAAAGGGTGCAAGGCAAAGG |  |
| 20 | **VEGF** (Vascular endothelial growth factor, vascular relating growth factor) | 260 | TCAGGGTTTCGGGAACCAGAC | TTCCGGGCTTGGCGATTTAG | Vascular relating growth factor |
| 21 | **HGF** (Hepatocyte growth factor, common elements to muscle and vascular growth) | 169 | TCAGCACCATCAAGGCAAGG | GATGGCACATCCACGACCAG |  |
| 22 | **PDGF-b** (Platelet-derived growth factor-b, vascular relating growth factor) | 271 | AGCCAAGACGCCTCAAGCTC | GGGTGAGGGAAGCACCATTG |  |
| 23 | **TGFb** (Transforming growth factor-b, vascular relating growth factor) | 195 | CCAAGGGCTACCATGCCAAC | ACTGCTCCACCTTGGGCTTG |  |
| 24 | **EGF** (Epidermal Growth Factor, common elements to muscle and nerve growth) | 137 | TGCGGATGGTACGAATGGTG | GTGGAATCCAGCAGCTTTGC |  |
| 25 | **FGF2** (basic fibroblast growth factor, common elements to muscle and nerve growth) | 161 | GCCAACCGGTACCTTGCTATG | TGCCCAGTTCGTTTCAGTGC |  |
| 26 | **HPRT** (hypoxanthine guanine phosphoribosyl transferase) | 111 | GCAAACTTTGCTTTCCCTGGTTAAG | CAACAAAGTCTGGCCTGTATCCA | House-keeping control gene |
