# Supplementary figures and images for: Reconstitution of the complete rupture in musculotendinous junction using skeletal muscle-derived multipotent stem cell sheet-pellets as a “bio-bond”
Source: PeerJ. 2016 Jul 19;4:e2231. doi: 10.7717/peerj.2231 (PMC4957990; doi:10.7717/peerj.2231)

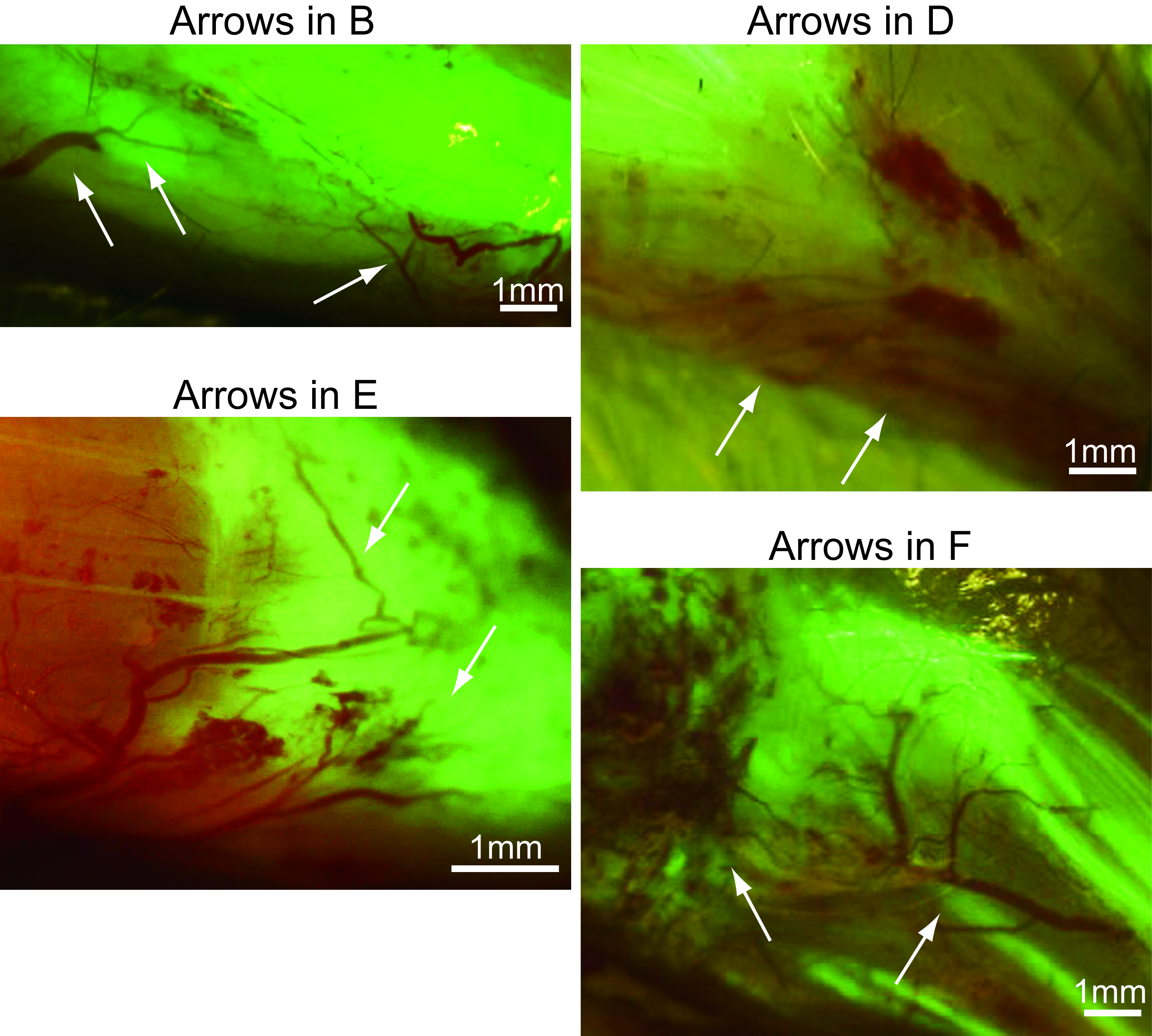

Supplement: Figure S1 — Interactions between blood vessels and GFP+ tissue are evident. [file peerj-04-2231-s003.jpg]

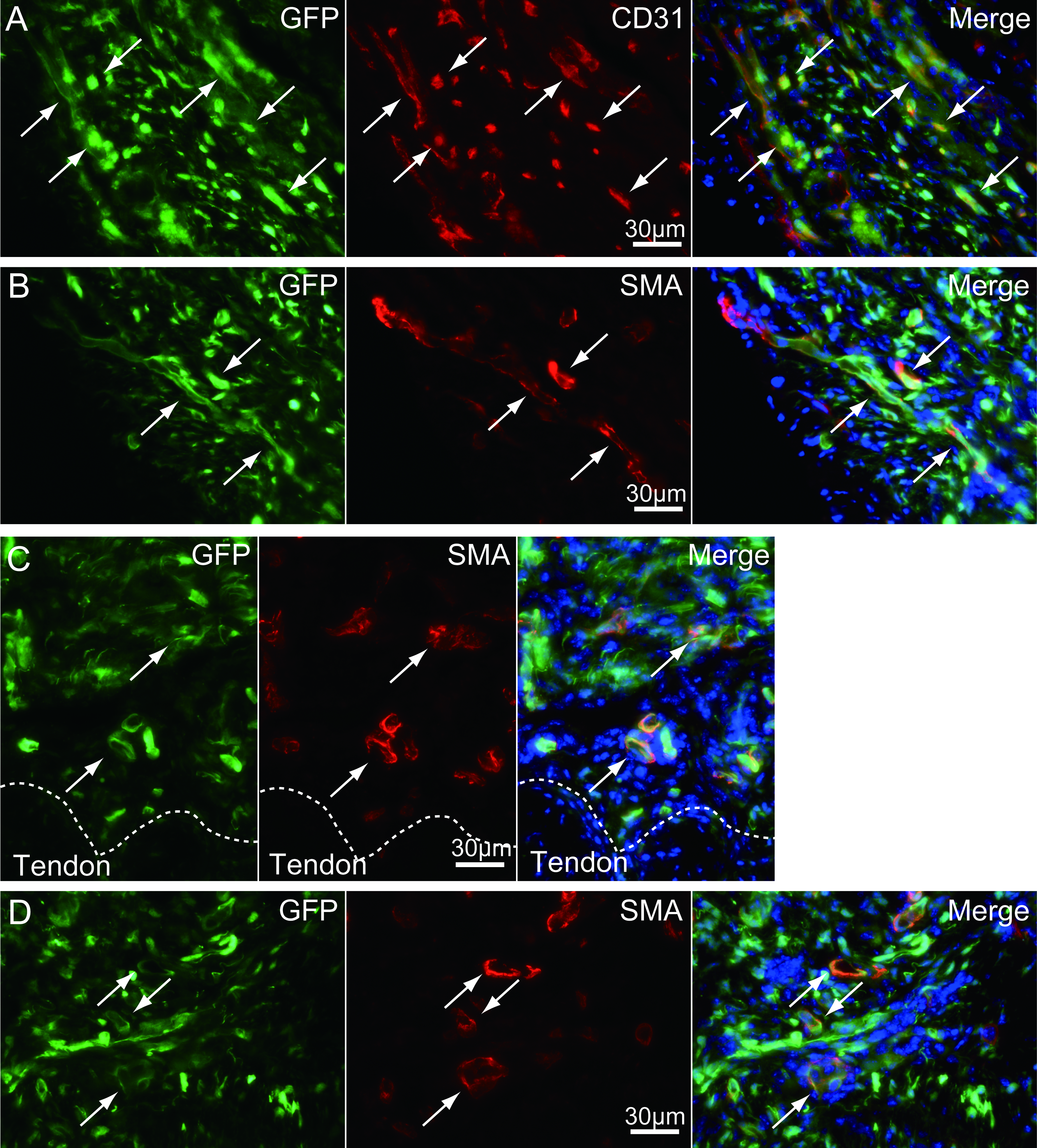

Supplement: Figure S2 — These are evidences of the differentiation of GFP+ donor cells into vascular endothelial cells (CD31) and vascular smooth muscle cells (SMA). [file peerj-04-2231-s004.jpg]

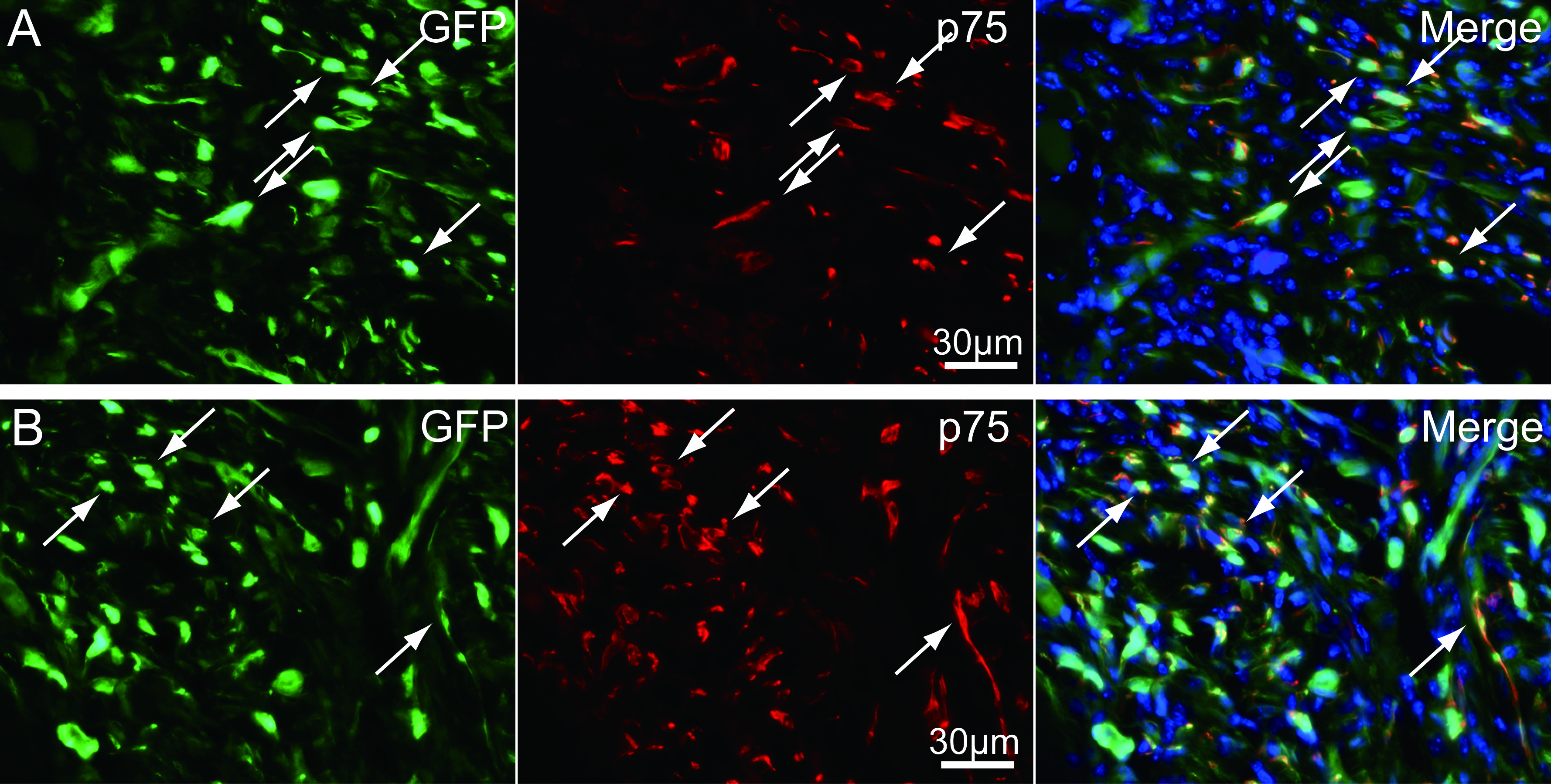

Supplement: Figure S3 — These are evidence of the differentiation of GFP+ donor cells into Schwann cells. [file peerj-04-2231-s005.jpg]
